# The Protective Effect of Esculentoside A on Experimental Acute Liver Injury in Mice

**DOI:** 10.1371/journal.pone.0113107

**Published:** 2014-11-18

**Authors:** Fang Zhang, Xingtong Wang, Xiaochen Qiu, Junjie Wang, He Fang, Zhihong Wang, Yu Sun, Zhaofan Xia

**Affiliations:** 1 Department of Burn Surgery, the Second Military Medical University affiliated Changhai Hospital, Shanghai, China; 2 Number 73901 Troop of PLA, Shanghai, China; 3 Department of General Surgery, 309th Hospital of PLA, Beijing, China; INRA, France

## Abstract

Inflammatory response and oxidative stress are considered to play an important role in the development of acute liver injury induced by carbon tetrachloride (CCl_4_) and galactosamine (GalN)/lipopolysaccharides (LPS). Esculentoside A (EsA), isolated from the Chinese herb phytolacca esculenta, has the effect of modulating immune response, cell proliferation and apoptosis as well as anti-inflammatory effects. The present study is to evaluate the protective effect of EsA on CCl_4_ and GalN/LPS-induced acute liver injury. In vitro, CCK-8 assays showed that EsA had no cytotoxicity, while it significantly reduced levels of TNF-α and cell death rate challenged by CCl_4_. Moreover, EsA treatment up-regulated PPAR-γ expression of LO2 cells and reduced levels of reactive oxygen species (ROS) challenged by CCl_4_. In vivo, EsA prevented mice from CCl_4_-induced liver histopathological damage. In addition, levels of AST and ALT were significantly decreased by EsA treatment. Furthermore, the mice treated with EsA had a lower level of TNF-α, Interleukin (IL)-1β and IL-6 in mRNA expression. EsA prevented MDA release and increased GSH-Px activity in liver tissues. Immunohistochemical staining showed that over-expression of F4/80 and CD11b were markedly inhibited by EsA. The western bolt results showed that EsA significantly inhibited CCl_4_-induced phosphonated IkBalpha (P-IκB) and ERK. Furthermore, EsA treatment also alleviated GalN/LPS-induced acute liver injury on liver enzyme and histopathological damage. Unfortunately, our results exhibited that EsA had no effects on CCl_4_-induced hepatocyte apoptosis which were showed by TUNEL staining and Bax, Caspase-3 and cleaved Caspase-3 expression. Our results proved that EsA treatment attenuated CCl_4_ and GalN/LPS-induced acute liver injury in mice and its protective effects might be involved in inhibiting inflammatory response and oxidative stress, but not apoptosis with its underlying mechanism associated with PPAR-γ, NF-κB and ERK signal pathways.

## Introduction

Liver is one of the most important internal organs in human body with multiple of functions such as detoxification, protein synthesis, and production of biochemicals necessary for digestion etc [1]. In addition, liver is also the most vulnerable organ attacked by chemical toxic agents [2]. Acute liver injury is usually referred as the rapid development of hepatocellular dysfunction with a poor prognosis. It frequently results from the induction of drugs, virus infection, toxins or hepatic ischemic-reperfusion injury, et al [3], [4]. CCl_4_ is a well-known hepatotoxin widely used to induce acute and chronic toxic liver injury in a wide range of laboratory animals [1], [5]. The toxicity of CCl_4_ results from its reductive dehalogenation by cytochrome P450 into the highly reactive free radical trichloromethyl radical (•CCl_3_). Free radicals probably activate Kupffer cells and mediate the hepatic inflammation process through producing TNF-α and other pro-inflammatory cytokines. In the presence of excess oxygen, •CCl_3_ can transform into trichloromethylperoxy radical CCl_3_OO•, another highly reactive species [2], [3]. This molecule can also decrease polyunsaturated fatty acids and cause lipid peroxidation, which contributes to severe cellular damage [6], [7]. Another classical animal model achieved by intraperitoneal injection of GalN/LPS has often been used for the study of acute liver injury. GalN acts as a sensitizing agent because it depletes hepatic stores of uridine triphosphate via the galactose pathway. Low doses of LPS cause modest inflammatory responses resulting in increased susceptibility to numerous hepatotoxic chemicals, combined with GalN. As a response, hepatocytes strive to clear LPS, which is followed by inflammatory response, oxidative stress and even hepatic necrosis [8].

**Figure 1:**
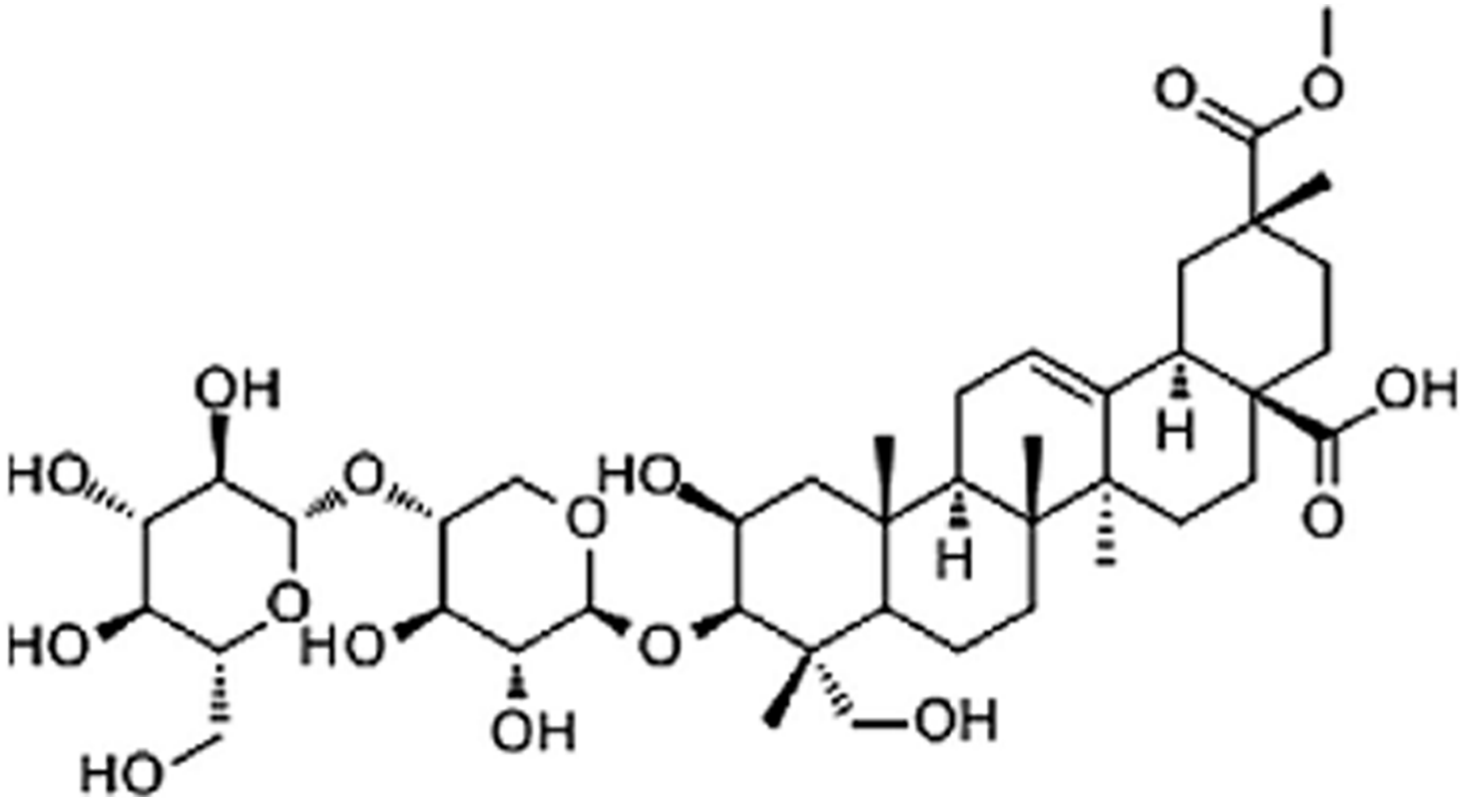
The molecular structure of Esculentoside A (EsA).

EsA is a saponin isolated from the root of Phytolacca esculenta, which is identified as 3-O-[b-D-glucopyranosyl-(1,4)-b-D-xylopyranosyl] phytolaccagenin (Fig. 1.) [9]. EsA has the effect of modulating immune response, cellular proliferation and apoptosis as well as anti-inflammatory effects in acute and chronic experimental models [10], [11]. Ju DW et al have demonstrated that EsA has the ability to inhibit pro-inflammatory cytokine production such as TNF-α, IL-1β, IL-2, IL-6 and prostaglandin E2 in several cell types [12], [13]. EsA is also referred to reduce the radiation-induced cutaneous and fibrovascular toxicities both in vivo and vitro [14]. Furthermore, EsA can also suppress inflammatory responses in LPS-induced acute lung injury through inhibiting the over-activity of NF-κB and mitogen activated protein kinase (MAPK) signal pathways [15]. However, it remains unclear whether EsA has a protective effect on CCl_4_ and GalN/LPS-induced acute liver injury, which might be associated with inhibiting inflammatory response and oxidative stress.

This is our first study to investigate the protective effect of EsA treatment on experimental acute liver injury. Therefore, we investigated the protective effect of EsA on experimental acute liver injury using both cell culture and animal experimental systems. In our study, we found that EsA treatment attenuated CCl_4_ and GalN/LPS-induced acute liver injury and its protective effect might be involved in inhibiting inflammatory response and oxidative stress, the underlying mechanism was associated with PPAR-γ, ERK and NF-κB signal pathways.

## Materials and Methods

### Cell culture and experimental animals

The human normal hepatocyte cell lines LO2 were kindly provided by the Eastern Hepato-Biliary Hospital, Second Military Medical University (SMMU), Shanghai. The cell line was cultured in Dulbecco's Modified Eagles Medium (DMEM) and 10% FBS at 37°C under a humidified atmosphere of 5% CO_2_.

Male C57Bl/6 mice (20–25g) were obtained from the Experimental Animal Center, SMMU, Shanghai. They were maintained under controlled conditions (23±3°C, 50±10% humidity and 12 h day/night rhythm) and fed standard laboratory chaw. All animal experiments were approved by Institutional Animal Care and Use Committee of SMMU in accordance with the Guide for Care and Use of Laboratory Animals published by U.S. National Institutes of Health (NIH) (publication No. 96-01).

### Material and reagents

EsA was kindly provided by the Department of Phytochemistry, College of Pharmacy, SMMU, Shanghai. Cell culture reagents were purchased from Invitrogen (Carlsbad, CA, USA). The ELISA kits of TNF-α was obtained from R&D Systems (Minneapolis, MN, USA). CCl_4_, GalN and LPS were purchased from Sigma (St. Louis, MO, USA). The rabbit monoclonal antibody Bax, Caspase-3, cleaved Caspase-3, ERK, P-ERK, IκB, P-IκB and GAPDH were purchased from Cell Signaling Technology Inc (Beverly, MA, USA). F4/80 and CD11b were purchased from Abcam (Cambridge, MA, USA). The rabbit monoclonal antibody PPAR-γ was purchased from Wanlei Biotechnology (Shenyang, China). The horseradish peroxidase econjugated goat anti-rabbit and goat anti-mouse antibodies were provided by Santa Cruz Biotechnology (Dallas, Texas, USA). CCK-8 assays were obtained from Dojindo Laboratories, Kumamoto, Japan. Trizol Reagent was purchased from Invitrogen (Carlsbad, CA, USA). MDA and GSH-px assays were obtained from Jiancheng Bioengineering Institute (Jiangsu, China). ROS assays were purchased from Beyotime institute of biotechnology (Jiangsu, China). All other chemicals used were of reagent grade.

### Experiment protocol

In vitro, LO2 cells were seeded at 5×10^3^ per well into 96 well microplate and incubated with DMEM and 10% FBS for 24 hours. The cell culture mediums were removed, different concentrations of EsA were added respectively (1.25 mg/L, 2.5 mg/L, 5 mg/L and 10 mg/L) and 70% CCl_4_ injury liquid was added as reported (n = 5 in each group) [16]. Parts of the cells were incubated for 4 hours, then 20 ul of CCK-8 solution was added to each well and incubated for another 4 hours at 37°C [17]. Other parts of LO2 cells were incubated with CCl_4_ and 5 mg/L EsA for 8 hours as above. Then the cell culture medium was sent for measurement of TNF-α. LO2 cells were observed for cell death rate using flow cytometry analysis. In order to evaluate the effect of EsA on the expression of PPAR-γ gene, LO2 cells were seeded at 5×10^6^ per well into 6 well microplate and incubated with DMEM and 10% FBS for 24 hours. PPAR-γ siRNA was cultured with LO2 cells for 24 hours according to the instruction manuals (F, 5′-CUGGCCUCCUUGAUGAAUATT-3′, R, 5′-UAUUCACAAGGAGGCCAGTT-3′ designed by Jima institute of biotechnology, Shanghai), then the cell culture mediums were removed, 2.5 mg/L EsA and 70% CCl_4_ injury liquid were added as above. After 4 hours, ROS assays, real-time PCR and western blot were performed.

In vivo, CCl_4_-induced acute liver injury was firstly performed to evaluate the effect of EsA treatment. Twenty-four mice were randomly divided into the following four groups (n = 6 in each group): (1) Control group, mice were injected i.p. with glycerol and distilled water (2∶3 v/v, 5 ml/kg) and olive oil (5 ml/kg). (2) EsA group, EsA was dissolved in glycerol and distilled water (2∶3 v/v) and given (i.p., 5 mg/kg) 30 minutes after administration of olive oil (i.p., 5 ml/kg). (3) Injury group, CCl_4_ mixed with olive oil (1∶9 v/v, 5 ml/kg) was injected i.p. for acute liver injury model, glycerol and distilled water (2∶3 v/v) were given (i.p., 5 ml/kg) 30 minutes later. (4) Injury + EsA group, EsA was dissolved in glycerol and distilled water (2∶3 v/v) and given (i.p., 5 mg/kg) 30 minutes after administration of CCl_4_ and olive oil as the Injury group.

GalN/LPS-induced acute liver injury model was performed as described [18]. The grouping was as above (n = 6 in each group): (1) Control group, mice were injected i.p. with glycerol and distilled water (2∶3 v/v, 5 ml/kg) and PBS water (5 ml/kg). (2) EsA group, EsA was dissolved in glycerol and distilled water as above and given (i.p., 5 mg/kg) 30 minutes after administration of PBS water (i.p., 5 ml/kg). (3) Injury group, GalN/LPS were dissolved in PBS water respectively and injected (i.p., GalN 800 mg/kg, LPS 5 ug/kg, PBS 5 ml/kg). After 30 minutes, glycerol and distilled water (i.p., 2∶3 v/v, 5 ml/kg) were given. (4) Injury + EsA group, EsA was given 30 minutes after GalN/LPS administration. The mice were sacrificed by an overdose of pentobarbital sodium (i.p., 20 mg/body weight) with blood sample and liver tissues collection 12 hours after CCl_4_ or GalN/LPS administration.

### Cytotoxicity assays *in* *vitro*

The EsA cytotoxicity to LO2 cells was measured by CCK-8 assays after CCl_4_ challenge. Cell viability was expressed as optical density (OD), which was proportional to the numbers of living cells. The absorbance of each well was measured by multiskan spectrum microplate reader (Biotek, USA) at 490 nm. The assays were performed as reported [19].

### Measurement of pro-inflammatory cytokines in LO2 culture medium

In order to test the anti-inflammatory effect of EsA in vitro, TNF-α in LO2 culture mediums were measured with a commercial ELISA kit following the instructions of the manufacturer.

### Flow cytometric analysis of cell death *in* *vitro*

To observe the effect of EsA on LO2 cell death rate induced by CCl_4_, LO2 cells treated with CCl_4_ were centrifuged at 1000 rpm for 10 minutes. The resulting pellet was suspended and adjusted to 1×10^6^ cells/ml in 10 ul propidium iodide (PI) buffer solution and 2 ul PI staining was performed to observe dead cells. The cells were analyzed by flow cytometry using a FACScan (Mitenyi Biotech, Germany). The measurement of cell death rate was obtained following the instructions of the manufacturer (Baihao biology CO., LTD, Beijing).

### Measurement of serum liver enzymes

To assess EsA toxicity to livers and the effect of EsA treatment for CCl**_4_** and GalN/LPS-induced acute liver injury, serum enzymes (AST and ALT) were measured by an automatic blood biochemical analyzer (7600-120, Hitachi High-Technologies Corp., Tokyo, Japan) using commercial kits [20].

### Hematoxylin and eosin staining (H&E)

For histological analysis, a portion of liver from the left lobe was fixed in 10% neutral-buffered formalin and embedded in paraffin. Sections of 5 µm thickness were affixed to slides, and stained with H&E. Finally, histopathological changes in the slices were observed with a light photomicroscope and were evaluated for pathological change using double blind method.

### Immunohistochemistry for CD11b and F4/80

Immunohistochemistry was performed with CD11b and F4/80 antibody by the methods as described previously [21]. Briefly, sections of liver tissues were deparaffinized and incubated with anti-mice-CD11b and F4/80 antibody overnight at 4°C. The sections were washed and then incubated with anti-rabbit-IgG. Secondary labeling was achieved by biotinylated rabbit anti-rat antibody. Horseradish peroxidase-conjugated avidin and brown-colored diaminobenzidine were used to visualize the labeling. Finally, the slides were counterstained with hematoxylin.

### Measurement of MDA levels and GSH-Px activity in vivo and ROS levels *in* *vitro*

MDA levels and GSH-Px activity in liver tissues were measured using commercial reagent kits (Jiancheng Bioengineering Institute, Jiangsu, China) according to the instruction manuals [22]. The measurement of ROS levels was performed as reported [23]. LO2 cells were stained with 2′,7′-dichlorofluorescein diacetate (DCFH-DA) for 30 minutes at 37°C in the dark, then the cells were washed three times with serum-free DMEM medium, the fluorescence intensity was detected with a multi-detection microplate reader with excitation at 488 nm and emission at 530 nm.

### Quantitative real-time PCR (SYBR Green method)

Total RNA was extracted from liver tissues and LO2 cells using Trizol. Quantitative real-time PCR was carried out using SYBR Green PCR Master Mix in a total volume of 10 ul on Step One Plus Real-Time PCR System (Applied Biosystems) as follows: 95°C for 30s, 40 cycles of 95°C for 15s, 60°C for 30s and 72°C for 35s. GAPDH and 18S were used as the reference genes. The relative levels of gene expression were represented as ΔCt = Ct gene−Ct reference, and the fold change of gene expression was calculated using the 2^−ΔΔCt^ method [24].

### Primer Sequences

•TNF-α (Mouse); F, 5′-TGTCTCAGCCTCTTCTCATT-3′,
R, 5′-AGATGATCTGAGTGTGAGGG-3′
•IL-1β (Mouse); F 5′- GCAGGCAGTATCACTCATTG-3′,
R, 5′-CACACCAGCAGGTTATCATC-3′
•IL-6 (Mouse); F, 5′-ATGAAGTTCCTCTCTGCAAGAGACT-3′,
R, 5′-CACTAGGTTTGCCGAGTAGATCTC-3′
•GAPDH (Mouse); F, 5′-AGAACATCATCCCTGCATCC-3′,
R, 5′- TCCACCACCCTGTTGCTGTA-3′
•PPAR-γ (Human); F, 5′-CAGGAAAGACAACAGACAAATCA-3′,
R, 5′-GGGGTGATGTGTTTGAACTTG-3′
•18S (Human); F, 5′-GGAAGGGCACCACCAGGAGT-3′,
R, 5′-TGCAGCCCCGGACATCTAAG-3′

### TUNEL stain

With the sections of liver tissues, terminal deoxynucleotidyl transferase dUTP nick end labeling (TUNEL) assay was performed using the In SituCell Death Detection Kit according to the manufacturer’s instructions. TUNEL-positive cells were counted by randomly selecting high-power fields [5].

### Western blot analysis

About 200 mg liver tissues or 5×10^6^ LO2 cells were homogenized in 1 ml tissue protein extraction reagent. The homogenates were centrifuged at 12,000 rpm for 20 minutes at 4°C, and the supernatants were collected. Protein concentration of the supernatants was determined by BCA protein assay kit. Western blot analysis was performed with ERK, P-ERK, Bax, Caspase-3, cleaved Caspase-3, IκB, P-IκB, PPAR-γ and GAPDH monoclonal antibodies as previously described [25].

### Statistical analysis

Statistical description was performed using SPSS 16.0 for windows (SPSS Inc., Chicago, USA). All data were expressed as means ± standard error of the mean (SEM). The statistical significance of differences between groups was analyzed using one-way analysis of variance and two-tailed Student t-test. p<0.05 was considered statistically significant.

## Results

### Measurement of EsA cytotoxicity and effects of EsA on CCl_4_-induced LO2 cell injury

**Figure 2:**
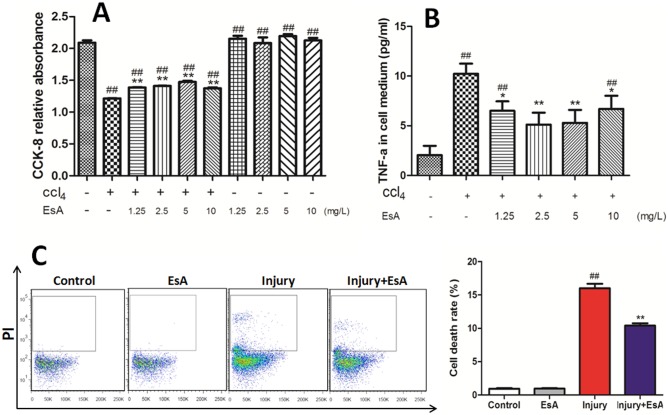
Measurement of EsA cytotoxicity and effects of EsA on CCl_4_-induced LO_2_ cell injury *in* *vitro*. The EsA cytotoxicity was measured using CCK-8 assays (A). Levels of TNF-α in LO2 culture mediums challenged by CCl_4_ increased to approximately 4-fold and which was dramatically prevented by EsA treatment, and the concentration of 2.5 mg/L reduced the level of TNF-α most obviously (B). The cell death rate was observed by flow cytometric analysis (C). The values presented are the means ± standard error of the mean (n = 5). ^##^P<0.01 versus the Control group. *P<0.05, **P<0.01 versus the Injury group.

The effect of EsA cytotoxicity to LO2 was assessed by CCK-8 assays. Absorbance in LO2 was not reduced by different concentrations of EsA (1.25, 2.5, 5 and 10 mg/L) compared with that in Control group (p>0.05). Furthermore, absorbance of LO2 treated with CCl_4_ and different concentrations of EsA was higher than that treated with CCl_4_ alone (p<0.01, Fig. 2. A). These results demonstrated that there was no cytotoxicity for EsA to LO2 cell lines at the concentration of lower than 10 mg/L and the viability of LO2 challenged by CCl_4_ might be effectively elevated by EsA treatment.

Levels of TNF-α in LO2 culture mediums challenged by CCl_4_ increased approximately 4-fold. However, different concentrations of EsA (1.25, 2.5, 5 and 10 mg/L) dramatically prevented TNF-α over-production, and EsA treatment reduced levels of TNF-α most obviously at the concentration of 2.5 mg/L (p<0.01, Fig. 2. B). The effect of EsA on cell death rate induced by CCl_4_ was observed by flow cytometric analysis. The cell death rate in Control group and EsA group were less than 1% respectively, the rate was more than 15% in the Injury group and which was significantly reduced by EsA treatment (p<0.01 Fig. 2. C).

**Figure 3:**
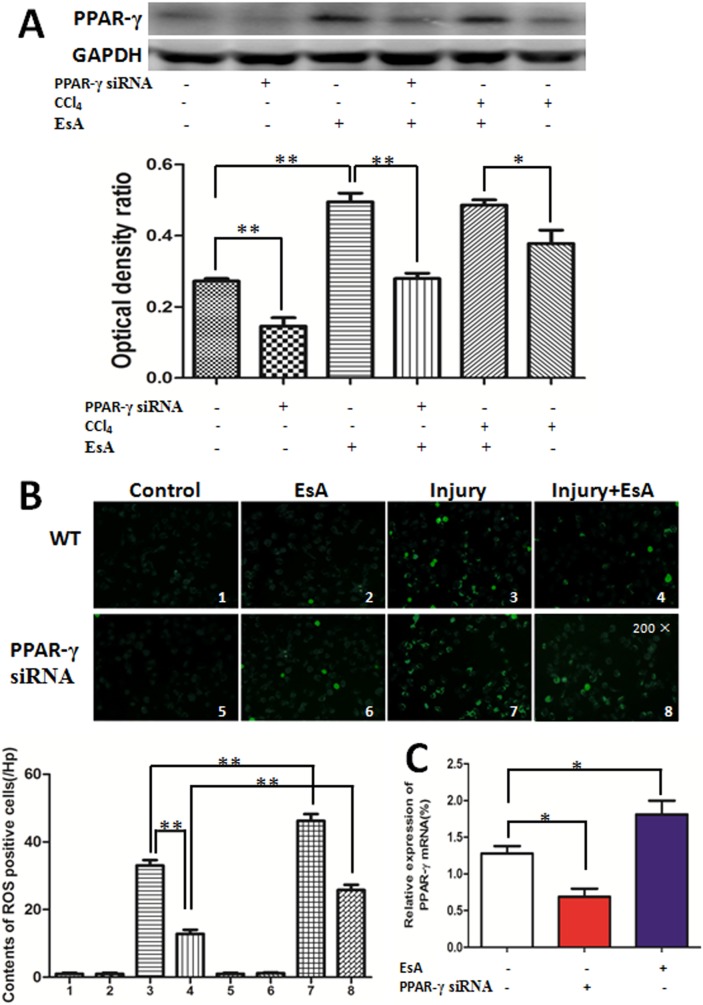
Effects of EsA on CCl_4_-induced LO_2_ cell injury and PPAR-γ expression. The treatment effects of EsA and protein expression of PPAR-γ were measured using western blot (A). Levels of ROS in LO2 cells challenged by CCl_4_ were shown (B Magnification, 200×). The mRNA expression of PPAR-γ was measured using quantitative real-time PCR (C). The values presented are the means ± standard error of the mean (n = 5). *P<0.05, **P<0.01.

As shown in Fig. 3., levels of cell ROS in the Control group and EsA group were both low, which were elevated rapidly after CCl_4_ challenge, and EsA treatment decreased levels of ROS significantly. However, the treatment effect of EsA on CCl_4_ injured cells was affected by PPAR-γ siRNA (p<0.01 Fig. 3. B). The western blot and real-time PCR results exhibited that PPAR-γ expression of LO2 cells were shut down significantly using siRNA technology, and expression of PPAR-γ was up-regulated obviously on cells treated with EsA (p<0.01 Fig. 3. A and p<0.05 Fig. 3. C). Moreover, western blot showed that EsA treatment could also up-regulate PPAR-γ expression of LO2 cells challenged by CCl_4_ (p<0.05 Fig. 3. A).

### EsA administration prevented CCl_4_-induced liver histopathological damage and hepatic dysfunction

**Figure 4:**
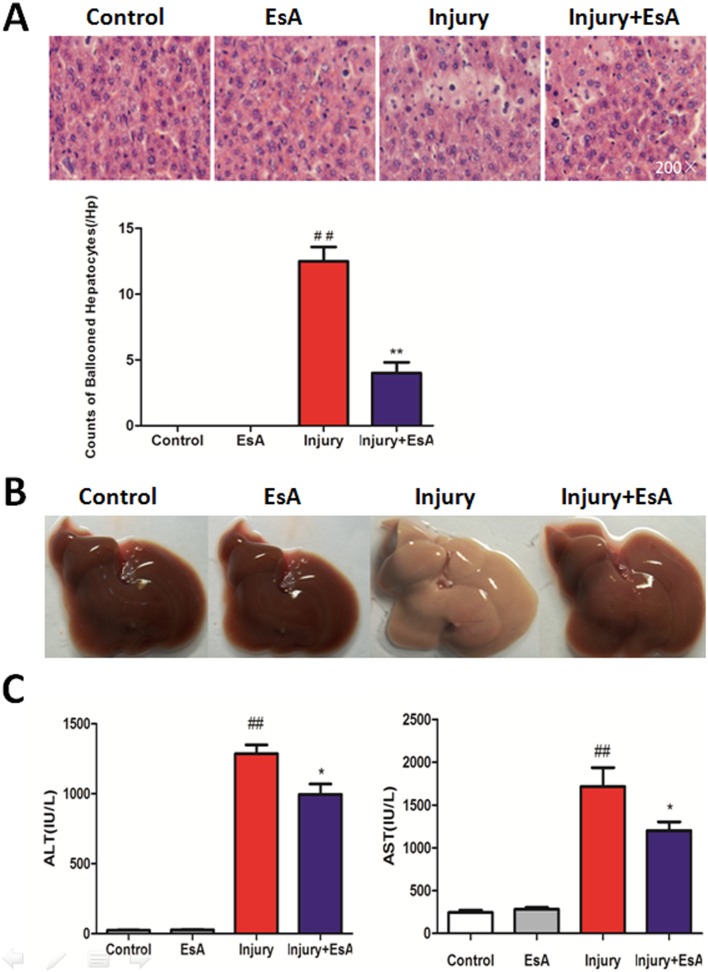
EsA protected against CCl_4_-induced histopathological damage and hepatic dysfunction. Hematoxylin and eosin staining (A Magnification, 200×) showed that livers in Injury group exhibited more ballooned hepatocytes than those in Control group and EsA group, and symptoms of those histopathological damage were significantly alleviated by EsA treatment (n = 6). Photographs of livers were taken 12 hours post-CCl_4_ injection, livers in Injury group turned white (B). Levels of AST and ALT increased obviously after CCl_4_ challenge. However, AST and ALT levels did not markedly increase in mice treated with EsA alone, and AST and ALT levels were significantly decreased with EsA treatment. (n = 6 C). The values presented are the means ± standard error of the mean. ^##^P<0.01 versus the Control group. *P<0.05, **P<0.01 versus the Injury group.

At 12 hours after CCl_4_-induced liver injury, liver sections showed normal cell morphology, with well-preserved cytoplasm and a clear plump nucleus in the Control and EsA group. However, significant anomalies of liver cells were observed in CCl_4_-injured mice, where many ballooned cells were exhibited, and symptoms of those histopathological damage were significantly alleviated by EsA treatment (p<0.01 Fig. 4. A). Furthermore, livers of the Injury group turned white at 12 hours after CCl_4_ injection, suggesting that CCl_4_ has induced severe liver cell injury. And livers treated with EsA seemed much better than those of the Injury group (Fig. 4. B).

To examine the effects of EsA on CCl_4_-induced hepatic dysfunction and toxicity of EsA to livers in mice, serum enzymes of liver function were measured. In the Control group, the ALT and AST activities were 24.17±8.01 and 244.17±68.88 IU/L respectively, and there were no markedly increase with a single injection with EsA, whereas the injection of CCl_4_ in mice led to a rapid increase of ALT and AST activities up to 1285.83±156.28 and 1798.33±440.18 IU/L respectively, with an obvious increases compared to those in the Control and EsA group (p<0.01). However, with the treatment of EsA, the serum activities were significantly decreased compared to the CCl_4_-intoxicated mice (p<0.05 Fig. 4. C).

### EsA treatment lessened oxidative stress in acute liver injury induced by CCl_4_

**Figure 5:**
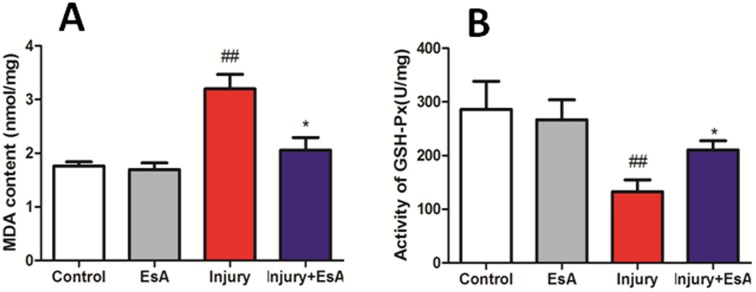
Effects of EsA on CCl_4_-induced liver oxidative stress. EsA treatment significantly decreased levels of MDA (A) and increased the activity of GSH-Px (B) compared with the Injury group. The values presented are the means ± standard error of the mean (n = 6). ^##^P<0.01 versus the Control group. *P<0.05 versus the Injury group.

In order to evaluate the effects of EsA treatment on oxidative stress induced by CCl_4_ in liver, we monitored MDA and GSH-Px. As shown in Fig. 5., EsA treatment significantly decreased MDA, a lipid peroxidative product of cell membranes, as indicated by a significant increase in MDA content from 1.76±0.18 and 1.69±0.31 nmol/mg in the Control and EsA group to 3.20±0.66 nmol/mg in the Injury group (p<0.05 Fig. 5. A). Furthermore, treament with EsA also increased the activity of GSH-Px compared with the Injury group markedly (p<0.05 Fig. 5. B) [26].

### Effects of EsA on pro-cytokine production and inflammatory cell infiltration following CCl_4_ challenge

**Figure 6:**
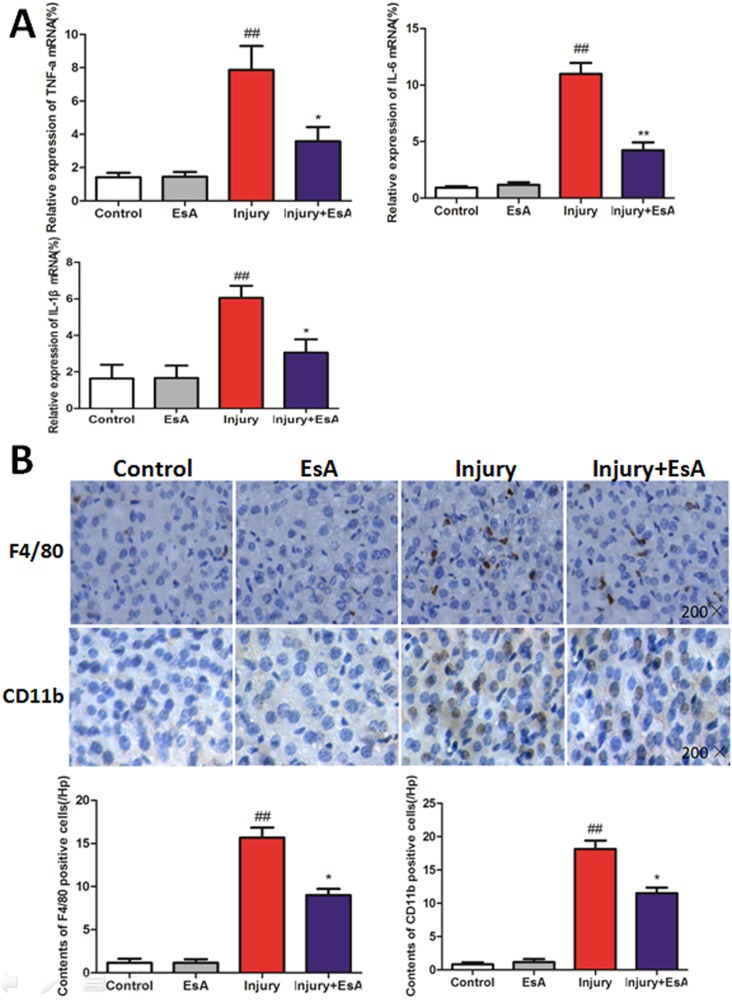
Effects of EsA on CCl_4_-induced liver inflammation. mRNA expression of TNF-a, IL-1β and IL-6 (A) and Immunohistochemical staining of F4/80 and CD11b cells (B) accumulating in liver tissues were determined at 12 hours post CCl_4_-induced acute liver injury (Magnification, 200×). The values presented are the means ± standard error of the mean (n = 6). ^##^P<0.01 versus the Control group. *P<0.05, **P<0.01 versus the Injury group.

As Fig. 6. showed that mice treated with EsA exhibited a lower mRNA expression of TNF-α, IL-1β and IL-6 compared with those in the Injury group (p<0.05 and p<0.01, Fig. 6. A). To characterize the inflammatory infiltration, liver sections were subjected to F4/80 antibody staining to identify the presence and distribution of macrophages, and CD11b antibody to identify neutrophils [27], [28]. Immunohistochemical staining showed that number of F4/80 and CD11b positive cells accumulating in liver sections was decreased by EsA treatment compared with that in Injury group (p<0.05, Fig. 6. B).

### Effects of EsA on ERK/NF-κB pathways activation in acute liver injury induced by CCl_4_

**Figure 7:**
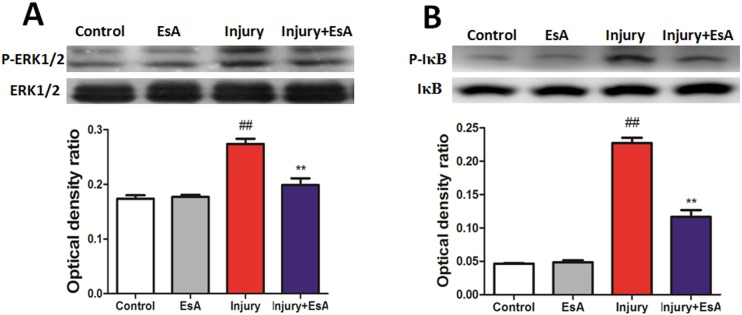
The underlying mechanism of EsA against CCl_4_-induced acute liver injury in mice. The activity of ERK (A) and IκB (B) were determined by western blot. Relative protein levels were quantified by densitometry and expressed as optical density ratio. The values presented are the means ± standard error of the mean (n = 6). ^##^ P<0.01 versus the Control group. **P<0.01 versus the Injury group.

MAPK families play an important role in CCl_4_-induced liver inflammatory response [29]. Herein, we tested the effects of EsA on ERK and NF-κB signal pathways in CCl_4_-induced acute liver injury. With the treatment of EsA, protein expression of P-ERK was significantly lower than that in Injury group. Furthermore, we found that EsA treatment also decreased over-expression of P-IκB induced by CCl_4_ compared with the Injury group (p<0.01, Fig. 7.).

### EsA treatment had no obvious effects on hepatocyte apoptosis induced by CCl_4_

**Figure 8:**
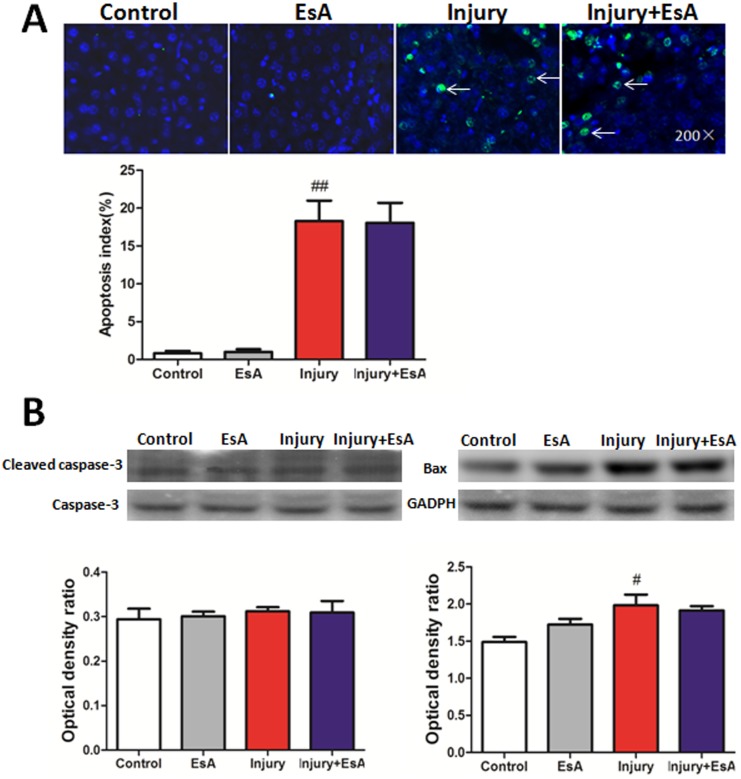
Effects of EsA on cell apoptosis at 12 hours post-CCl_4_ injection. Liver tissues sections were stained with TUNEL method (Magnification, ×200). There were no obvious difference for rates of positive TUNEL stained cells between the Injury and Injury+EsA groups (A). The activity of Bax, Caspase-3 and cleaved Caspase-3 were determined by western blot. Relative protein levels were quantified by densitometry and expressed as optical density ratio (B). The values presented are the means ± standard error of the mean (n = 6). ^#^P<0.05, ^##^P<0.01 versus the Control group.

As Fig. 8. showed that treatment with EsA had no obvious effects on TUNEL stain and protein expression of Bax, Caspase-3 and cleaved Caspase-3 compared with the Injury group (p>0.05, Fig. 8.).

### EsA protected against GalN/LPS-induced liver histopathological damage and hepatic dysfunction

**Figure 9:**
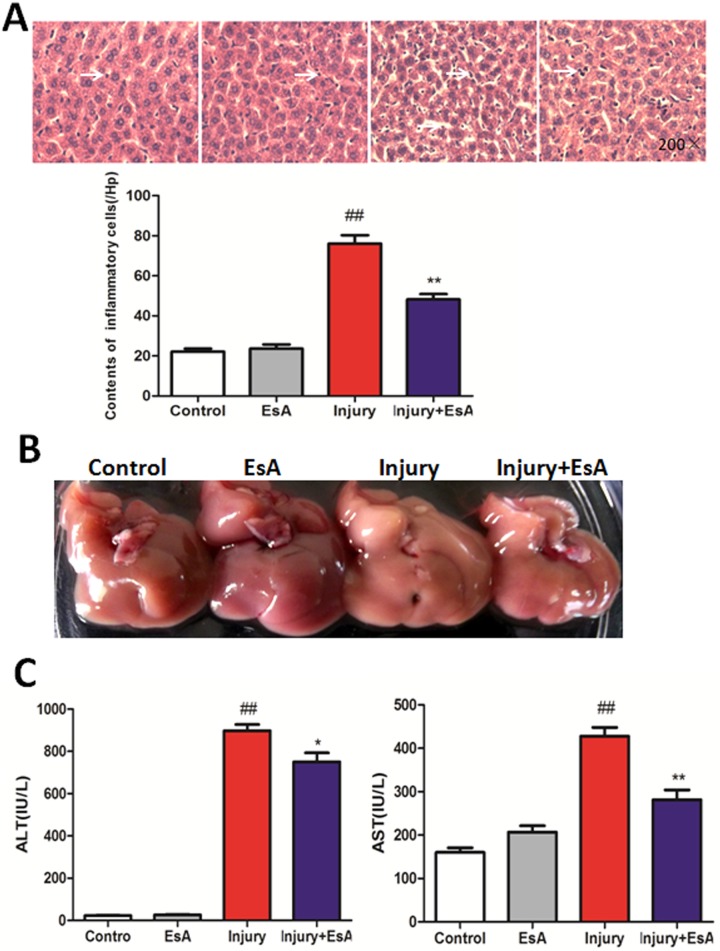
EsA protected against GalN/LPS-induced histopathological damage and hepatic dysfunction. Hematoxylin and eosin staining (A Magnification, 200×) showed that livers in Injury group exhibited more inflammatory cells than those in Control group and EsA group, which were significantly alleviated by treatment of EsA (n = 6). Liver photographs were taken 12 hours post-GalN/LPS administration, and livers in Injury group turned white (B). Levels of AST and ALT increased obviously after GalN/LPS challenge, and which were significantly decreased with EsA treatment (n = 6 C). The values presented are the means ± standard error of the mean. ^##^P<0.01 versus the Control group. *P<0.05, **P<0.01 versus the Injury group.

At 12 hours after GalN/LPS-induced liver injury, abnormal cell morphology and many inflammatory cells were observed in the Injury group compared with those in Control group and EsA group. However, symptoms of these histopathological damage were significantly alleviated by EsA treatment (p<0.01 Fig. 9. A). And livers of the Injury group turned white at 12 hours after GalN/LPS administration, suggesting severe liver cell injury has been induced by GalN/LPS, and EsA treatment alleviated these liver damage (Fig. 9. B). Serum enzymes were measured to evaluate effects of EsA treatment on GalN/LPS-induced hepatic dysfunction in mice. The injection of GalN/LPS led to a rapid increase of ALT and AST activities up to 897.5±71.75 and 427.5±50.07 IU/L respectively, with an obvious increases compared to those in the Control and EsA group (p<0.01). However, with the treatment of EsA, the serum activities of ALT and AST were significantly decreased compared to the mice in the Injury group (p<0.05 and p<0.01 Fig. 9. C).

## Discussion

The present study demonstrated that treatment with EsA could protect the liver from CCl_4_ and GalN/LPS-induced acute injury in both cell culture and animal experimental systems. We found that EsA treatment significantly reduced hepatic enzymes release, pro-inflammatory cytokine production, inflammatory cells infiltration and oxidative stress damage. Importantly, EsA was proved to have no cytotoxicity in vitro. This must be a preliminary study in demonstrating that EsA treatment can ameliorate CCl_4_ and GalN/LPS-induced acute liver injury.

Wu et al. referred that haemolytic activity was the main toxicity of EsA and the high concentration might have cytotoxicity [16]. Therefore, we firstly measured cytotoxicity for EsA to LO2 in vitro by CCK-8 assays, we found that there was no cytotoxicity for EsA to LO2 at the concentration of lower than 10 mg/L and the viability of LO2 treated with CCl_4_ might be effectively promoted by EsA treatment. Therefore, we started investigating the protective effect of EsA in CCl_4_ and GalN/LPS-induced acute liver injury.

As the acute liver injury induced by CCl_4_ and GalN/LPS was characterized with liver dysfunction and cell morphology deterioration, the liver histopathological changes and liver function were investigated. In our study, it was found that EsA treatment could significantly mitigate liver histopathological changes as evidenced by H&E staining. Meanwhile, high levels of ALT and AST challenged by CCl_4_ and GalN/LPS, which are direct indicators of hepatic function and correlated with the severity of liver injury, were markedly prevented by treatment with EsA. Our results showed that EsA treatment could lessen hepatic dysfunction and cell morphology deterioration in acute liver injury induced by CCl_4_ and GalN/LPS.

Inflammatory cell infiltration and inflammatory response were proved to be involved in the process of CCl_4_-induced acute chemical liver injury [30]. In our study, F4/80 (marker for mature mouse macrophages) and CD11b antibodies (marker of neutrophil activation) were used to stain inflammatory cells in liver sections and we found that EsA treatment obviously reduced inflammatory infiltration compared with that in Injury group [27], [28]. Previous studies have showed that EsA could decrease both extracellular and cellular TNF-α in a dose dependent manner at concentrations of higher than 1 µmol/L [31]. Similarly, levels of pro-inflammatory cytokines, which are direct indicators of inflammatory response, were also significantly reduced by EsA administration. In our study, EsA treatment also showed obvious effect of preventing the over-production of TNF-α in LO2 cell medium challenged by CCl_4_. However, we found that levels of TNF-α were reduced not in a dose dependent manner, and the effect of EsA treatment was best at concentrations of 2.5 mg/L. To explore the underlying anti-inflammatory mechanism of EsA on CCl_4_-induced acute liver injury, NF-κB and ERK signal pathways were investigated. Previous works have proved that NF-κB regulated the expression of multiple genes involved in the early inflammatory response, which played a central role in the pathology of acute liver injury and inflammation [32]. Zhong et al. exhibited that EsA-treatment decreased the NF-κB expression in LPS-induced acute lung injury in mice [15]. We found that the active NF-κB signal pathway challenged by CCl_4_ was inhibited by EsA treatment, similarly with the ERK. We also found that EsA treatment could up-regulate PPAR-γ gene expression of LO2 cells, and the low expression of PPAR-γ affected the treatment effects of EsA. Thus, the beneficial effect of EsA may be partly due to attenuating inflammatory response in CCl_4_ and GalN/LPS-induced acute liver injury via PPAR-γ, ERK and NF-κB signal pathways.

Oxidative stress has been postulated as major molecular mechanisms in acute liver injury induced by CCl_4_ [33], [34]. Yu et al. referred that the levels of MDA and GSH-Px were associated with CCl_4_-induced liver oxidative stress injury [35]. Increased MDA, a lipid peroxidative product of cell membranes, was prevented by EsA treatment in our study [36]. Furthermore, EsA treatment led to an obvious increase in GSH-Px activity, compared with the Injury group. Overall, the protective effect of EsA may be partly due to attenuating oxidative stress in acute liver injury.

Xiao et al. showed that EsA has the positive curative effect on autoimmunity in a mouse model through the acceleration of thymocyte apoptosis [37]. Hu et al. demonstrated that EsA affected pro-apoptotic genes included Fas, p53, redox metabolism, calcium and glucocorticoid-associated apoptosis signals [11]. Therefore, we investigated the effect of EsA on mitochondrial apoptotic pathways including Bax, Caspase-3 and cleaved Caspase-3. But our works reflected that EsA treatment had no effect on CCl_4_-induced hepatocyte apoptosis, which were proved by the TUNEL staining and apoptosis–associated protein expression.

In summary, treatment with EsA attenuated CCl_4_ and GalN/LPS-induced acute liver injury in mice, and the protective mechanism might be involved in inhibiting inflammatory response and oxidative stress, but not apoptosis. Accordingly, EsA may have potential applications as a supportive treatment for acute liver injury due to its unique advantages.

## Supporting Information

Figure S1 **The primary data for EsA cytotoxicity and treatment effect of EsA in vitro.**
(XLS)

Figure S2 **The primary data for EsA treatment on CCl_4_-induced LO2 cell injury and PPAR-γ expression.**
(XLS)

Figure S3 **The primary data for protection of EsA against CCl_4_-induced histopathological damage and hepatic dysfunction.**
(XLS)

Figure S4 **The primary data for EsA treatment on CCl_4_-induced liver oxidative stress in mice.**
(XLS)

Figure S5 **The primary data for EsA treatment on CCl_4_-induced liver inflammation in mice.**
(XLS)

Figure S6 **The primary data for underlying mechanism of EsA against CCl_4_-induced acute liver injury.**
(XLS)

Figure S7 **The primary data for effects of EsA on cell apoptosis after CCl_4_ injection.**
(XLS)

Figure S8 **The primary data for protection of EsA against GalN/LPS-induced histopathological damage and hepatic dysfunction.**
(XLS)
